# Predicting mortality after acute coronary syndromes in people with chronic obstructive pulmonary disease

**DOI:** 10.1136/heartjnl-2016-309359

**Published:** 2016-05-13

**Authors:** Kieran J Rothnie, Liam Smeeth, Neil Pearce, Emily Herrett, Adam Timmis, Harry Hemingway, Jadwiga Wedzicha, Jennifer K Quint

**Affiliations:** 1 Respiratory Epidemiology, Occupational Medicine and Public Health, National Heart and Lung Institute, Imperial College London, London, UK; 2 Faculty of Epidemiology and Population Health, London School of Hygiene and Tropical Medicine, London, UK; 3 Farr Institute of Health Informatics Research, London, UK; 4 Barts NIHR Biomedical Research Unit, Queen Mary University of London, London, UK; 5 Department of Epidemiology and Public Health, University College London, London, UK; 6 Airway Disease, National Heart and Lung Institute, Imperial College London, London, UK

## Abstract

**Objective:**

To assess the accuracy of Global Registry of Acute Coronary Events (GRACE) scores in predicting mortality at 6 months for people with chronic obstructive pulmonary disease (COPD) and to investigate how it might be improved.

**Methods:**

Data were obtained on 481 849 patients with acute coronary syndrome admitted to UK hospitals between January 2003 and June 2013 from the Myocardial Ischaemia National Audit Project (MINAP) database. We compared risk of death between patients with COPD and those without COPD at 6 months, adjusting for predicted risk of death. We then assessed whether several modifications improved the accuracy of the GRACE score for people with COPD.

**Results:**

The risk of death after adjusting for GRACE score predicted that risk of death was higher for patients with COPD than that for other patients (RR 1.29, 95% CI 1.28 to 1.33). Adding smoking into the GRACE score model did not improve accuracy for patients with COPD. Either adding COPD into the model (relative risk (RR) 1.00, 0.94 to 1.02) or multiplying the GRACE score by 1.3 resulted in better performance (RR 0.99, 0.96 to 1.01).

**Conclusions:**

GRACE scores underestimate risk of death for people with COPD. A more accurate prediction of risk of death can be obtained by adding COPD into the GRACE score equation, or by multiplying the GRACE score predicted risk of death by 1.3 for people with COPD. This means that one third of patients with COPD currently classified as low risk should be classified as moderate risk, and could be considered for more aggressive early treatment after non-ST-segment elevation myocardial infarction or unstable angina.

## Introduction

Accurate prediction of risk of death after acute coronary syndromes (ACS) is important for prognostication and decision making about treatment, as individuals at higher risk of death after ACS benefit most from early aggressive treatment.^1^ ^2^ Early and accurate assessment of future risk allows clinicians to identify patients who might benefit most from therapies and to avoid unnecessary treatment for those who are less likely to benefit.

Global Registry of Acute Coronary Events (GRACE) scores are used internationally to predict the probability of death at 6 months after admission to hospital for ACS. They have been developed and validated in several different settings.^3–7^ The predicted risk of death can be used to stratify patients into low (<3%), moderate (3%–6%) and high (>6%) risk of death at 6 months after ACS. Current guidelines recommend that those classified as moderate and high risk of death using the GRACE score should receive more aggressive early therapy after non-ST-segment elevation myocardial infarction (non-STEMI) or unstable angina.^8^ ^9^

People with chronic obstructive pulmonary disease (COPD) have a higher risk of myocardial infarction (MI) than those without COPD, and cardiovascular disease is an important cause of death in people with COPD. In addition, COPD is very common in people with MI, with prevalence ranging from 10% to 17%.^10^ ^11^ Several studies have also found an increased risk of death after MI in people with COPD compared with those without COPD.^10^ ^12^ ^13^ Previous work^14^ has shown that, after adjusting for confounders, even though people with COPD have a higher mortality at 6 months after discharge than non-COPD patients, they are less likely to receive angiography in hospital after a non-STEMI, or to receive secondary prevention drugs after any MI. One of the reasons for this may be that GRACE scores may not predict risk of death in patients with COPD as well as they do in patients without COPD.

Using data from the UK Myocardial Ischaemia National Audit Project (MINAP) registry, we investigated whether GRACE scores performed as well in people with COPD as they do in people without COPD, and how they might be improved for people with COPD.

## Methods

### Data source

MINAP is a UK registry of all admissions for ACS to hospitals in England and Wales. The following variables were collected which are needed for the equation for 6-month mortality (post admission): age, heart rate, systolic blood pressure, creatinine, heart failure, cardiac arrest at admission, ST-segment deviation and elevated cardiac enzymes.^15^ Vital status is available through linkage with the Office of National Statistics (ONS) mortality data.

We included all patients with a diagnosis of STEMI from January 2003 to June 2013, or non-STEMI or unstable angina from January 2004 to December 2012. Diagnosis of STEMI, non-STEMI and unstable angina were based on physician diagnosis and records of ECG and cardiac biomarker findings. Records were excluded if they did not have a patient unique identifier; if patients had missing values for presence of obstructive airway disease or smoking history; or if ONS mortality data were missing.

We identified COPD in MINAP using a strategy previously validated in MINAP data linked with primary care.^14^ Briefly, we used the obstructive airway disease indicator and a smoking history (ex-smoker or current smoker) to identify COPD, and this identified COPD with a misclassification rate of <10%.

### Statistical methods

#### GRACE scores

GRACE scores and predicted risks of death at 6 months were constructed using published nomograms for the Fox model.^16^ Values available from nomograms were used to construct algorithms to score patients and to convert these to predicted risk death. As Killip class is not recorded in MINAP, we used a previously validated^17^ method to score patients based on Killip class of heart failure by using in-hospital prescription of diuretics as a proxy.

We estimated the observed and GRACE score predicted risks of death at 6 months and compared these between people with and without COPD. We estimated the Mantel–Haenszel risk ratio averaged over the GRACE score deciles to estimate the average relative risk for death at 6 months after admission for patients with COPD with the same GRACE score as non-COPD patients. If GRACE scores work equally well in patients with COPD and patients without COPD, then the risk ratio would be 1. A risk ratio of <1 would suggest that GRACE scores overestimate the risks of death in patients with COPD after admission; a risk ratio of >1 would suggest that GRACE scores underestimate the risks of death in patients with COPD. We also compared the risk of death for people with diabetes with those who do not have diabetes, adjusted for GRACE score predicted risk of death.

We then investigated the observed risk of death between patients with COPD and without COPD within GRACE score predicted levels of risk (0%–3% low, 3%–6% moderate and >6% high).

We explored the extent of and possible reasons for missingness of GRACE score variables and performed a multiple imputation analysis (see details in online supplementary material).

10.1136/heartjnl-2016-309359.supp1 Supplementary data

#### Model modifications

We investigated several strategies for improving GRACE scores for people with COPD. We prespecified three potential modifications to the GRACE models which might improve their accuracy for patients with COPD: (1) adding COPD into the models as a risk factor, (2) adding smoking history into the models as a risk factor and (3) multiplying the predicted risk of death for patients with COPD by the RR for risk of death for patients with COPD compared with non-COPD patients after adjusting for GRACE score predicted risk of death.

For the approaches which involved adding new variables to the models (smoking and COPD), we had to respecify the GRACE models. We did this by building logistic regression models which included all of the GRACE variables (with or without smoking or COPD) with death at 6 months as the outcome and used these to predict risk of death. As an internal validation procedure, we also bootstrapped the logistic regression models with 100 reps each, and compared the parameter estimates with those from the main analysis.

To assess which models performed best, we calculated the Mantel–Haenszel risk ratios to compare the risk of death at 6 months between patients with COPD and without COPD adjusting for predicted risk of death for the model in question. We also calculated C-statistics and Hosmer–Lemeshow goodness of fit tests. Strategies involving multiplication of risk for patients with COPD using the existing GRACE model were compared with the existing GRACE model. To make a fair comparison, models which involved adding other variables (smoking or COPD) were compared with our models which included all of the GRACE variables. To assess how well each model stratified risk, we also plotted the proportion of all deaths by deciles of predicted risk of death at 6 months for the normal GRACE model and for modifications. We calculated how many people would be reclassified in terms of risk level (low, moderate or high) for each modification, we also performed this analysis stratified by type of ACS. Finally, we also calculated the continuous net reclassification improvement (NRI) statistic^18^ for adding COPD to the GRACE score model.

### Ethics

This study was approved by London School of Hygiene and Tropical Medicine (LSHTM) Observational Ethics Committee (6468) and the MINAP academic group (13-MNP-07).

## Results

### Patient characteristics

**Figure 1:**
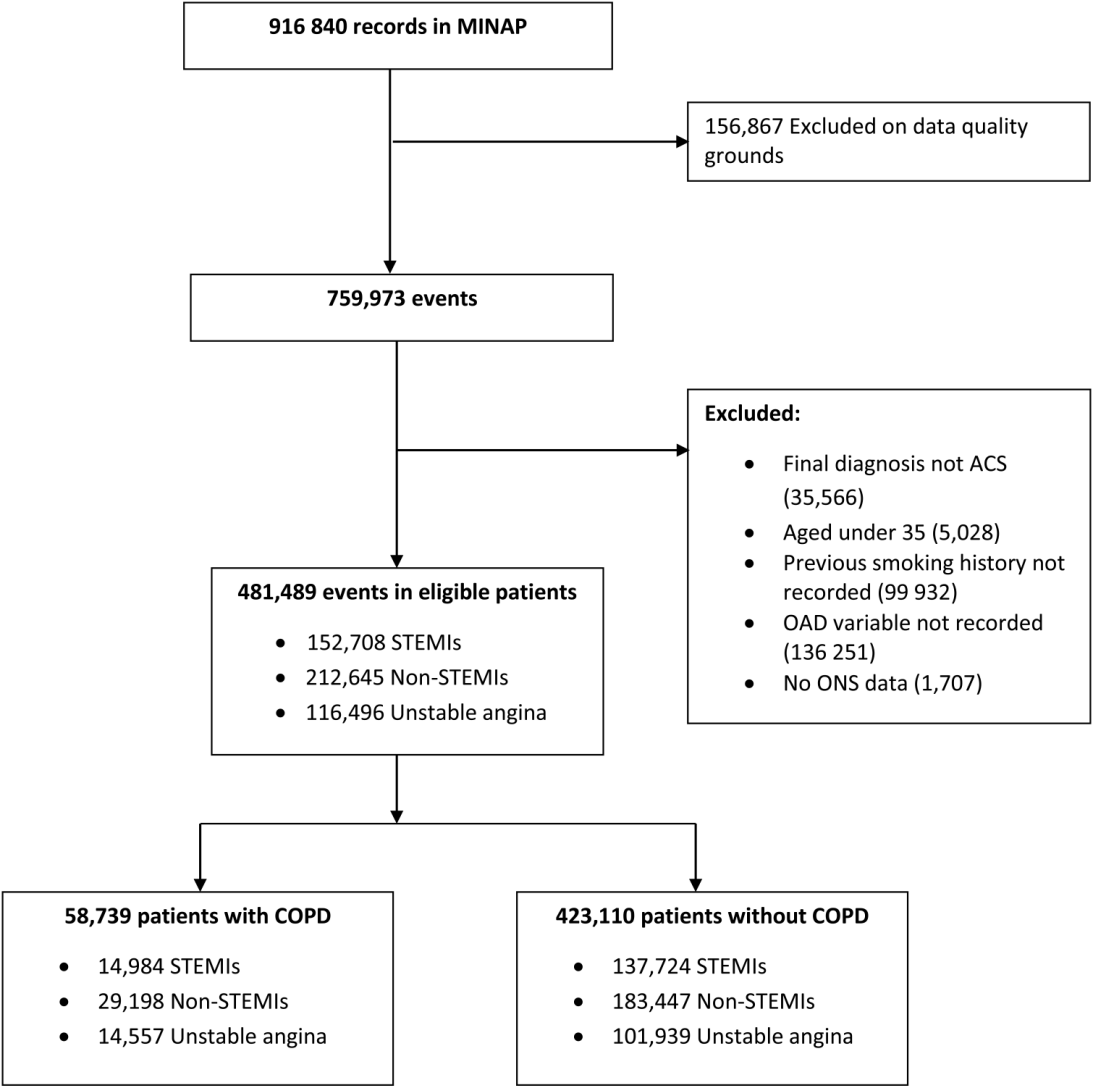
Flow of participants through the study. ACS, acute coronary syndromes; COPD, chronic obstructive pulmonary disease; MINAP, Myocardial Ischaemia National Audit Project; non-STEMI, non-ST-elevation myocardial infarction; OAD, obstructive airway disease; ONS, Office of National Statistics.

In total, 481 489 patients with ACS were included, of whom 58 739 (12.2%) had COPD (figure 1). Characteristics of patients with COPD and without COPD are shown in table 1. In terms of mortality, patients with COPD were more likely to have died by 6 months after admission compared with those without COPD (17.7% compared with 11.6%). Patients with COPD, on average also had higher GRACE score predicted risk of death than those without COPD (14.0% (SD, 12.7) compared with 11.7% (SD, 12.3)).

**Table 1:** Characteristics of patients included in the analysis

Characteristic	Non-COPD	COPD
Age group (n=481 489)		
<55	79 603 (18.8%)	6575 (11.2%)
55–64	92 446 (21.8%)	10 858 (18.5%)
65–74	101 654 (24.0%)	17 402 (29.6%)
75–84	100 660 (23.8%)	18 011 (30.7%)
≥85	48 747 (11.5%)	5893 (10.0%)
Sex (n=481 489)		
Male	285 502 (67.5%)	37 135 (63.2%)
Female	137 608 (32.5%)	21 604 (36.8%)
Diagnosis (n=481 489)		
STEMI	137 724 (32.6%)	14 984 (25.5%)
Non-STEMI	183 447 (43.4%)	29 198 (49.7%)
Unstable angina	101 393 (24.1%)	3136 (24.8%)
Previous MI (n=478 530)	79 733 (18.9%)	14 485 (25.1%)
Previous angina (n=477 494)	107 991 (25.7%)	19 962 (34.7%)
Previously treated hyperlipidaemia (n=467 096)	135 236 (32.9%)	18 573 (33.2%)
Previously treated hypertension (n=477 515)	201 174 (47.9%)	28 256 (49.0%)
Peripheral vascular disease (n=473 652)	17 216 (4.1%)	4182 (7.4%)
Cerebrovascular disease (n=476 863)	31 563 (7.5%)	5858 (10.3%)
Chronic renal failure (n=476 351)	17 368 (4.1%)	3697 (6.5%)
Chronic heart failure (n=476 324)	18 216 (4.3%)	4955 (8.7%)
Previous percutaneous coronary intervention (n=472 614)	29 077 (7.0%)	4256 (7.5%)
Previous coronary artery bypass graft (n=473 891)	22 567 (5.4%)	3320 (5.8%)
Smoking history (n=481 849)		
Never smoker	142 254 (33.6%)	0 (0%)
Ex-smoker	151 560 (35.8%)	35 103 (59.8%)
Current smoker	129 296 (30.6%)	23 636 (40.2%)
Raised cardiac markers* (n=481 849)	365 730 (91.8%)	51 206 (92.2%)
ST-segment deviation* (n=413 253)	221 205 (60.7%)	27 165 (55.3%)
Use of diuretic in hospital* (n=481 849)	93 116 (22.0%)	19 069 (32.5%)
Mean heart rate* (n=433 721)	80.2±21.9	87.2±23.7
Mean systolic blood pressure* (n=432 854)	139.9±28.6	138.2±29
Mean serum creatinine* (n=287 893)	101±56.6	103.4±58.3

*Mean±SD.

COPD, chronic obstructive pulmonary disease; MI, myocardial infarction; non-STEMI, non-ST-segment elevation myocardial infarction.

### GRACE score performance

The Mantel–Haenszel pooled risk ratio comparing risks of death for patients with COPD with those without COPD after adjusting for GRACE score predicted risk of death was 1.30 (95% CI 1.27 to 1.33). Observed and predicted mortality for patients with COPD and without COPD, split by deciles of GRACE score predicted risk of death, is presented in table 2. These results stratified by year of admission are presented in the online supplementary table S1. People with diabetes also had a higher risk of death than those without diabetes with the same GRACE score predicted risk of death; however, this was lower than for people with COPD (RR 1.14, 95% CI 1.12 to 1.16).

**Table 2:** Predicted and observed mortality using the normal GRACE model

GRACE predicted risk decile	Average predicted mortality (%)	Observed mortality— non-COPD (%)	Observed mortality— COPD (%)
1	1.3	0.6	0.8
2	2.5	1.3	2.4
3	4.0	2.4	4.6
4	5.0	3.2	6.4
5	6.5	4.5	7.4
6	8.9	7.1	12.2
7	12.4	10.7	17.1
8	17.2	16.7	21.9
9	26.6	27.2	32.1
10	48.4	44.0	47.9

COPD, chronic obstructive pulmonary disease; GRACE, Global Registry of Acute Coronary Events.

### Model modifications

Findings from model modifications are displayed in table 3. Compared with the MINAP-derived GRACE score model using the original variables, the model including COPD as a risk factor resulted in better predictions for patients with COPD. Including smoking history as a risk factor in the model did not result in better predictions for patients with COPD. Bootstrapped results did not differ from the main analysis. Multiplying the GRACE score predicted risk of death by the RR for risk of death for patients with COPD adjusted for GRACE score predicted risk of death (1.3) resulted in a very close approximation to adding COPD into the model as a risk factor. C-statistics were improved for the model which multiplied the risk of death for patients with COPD by 1.3 and the model which included COPD as a risk factor. Adding smoking to the GRACE score model did not significantly change the C-statistic. Hosmer–Lemeshow statistics showed that all models tested had adequate calibration.

**Table 3:** Predictive ability of modifications to the GRACE score in patients with COPD

Method for obtaining predicted risk of death	M-H pooled RR (95% CI) for death at 6 months adjusted for predicted risk of death	C-statistics	Hosmer–Lemeshow p value
*Normal GRACE score (comparator for 1)*	1.29 (1.28 to 1.33)	0.8166	>0.999
1. Normal GRACE score—multiply risk of death by 1.3 for patients with COPD	0.99 (0.96 to 1.01)	0.8181 (p<0.001)*	>0.999
*MINAP-derived GRACE score (comparator for 2–3)*	1.23 (1.20 to 1.26)	0.8322	>0.999
2. MINAP-derived GRACE score+smoking	1.20 (1.17 to 1.23)	0.8323 (p=0.274)*	>0.999
3. MINAP-derived GRACE score+COPD	1.00 (0.94 to 1.02)	0.8333 (p<0.001)*	>0.999

*p Values compare the C-statistics for the modified models with either the normal GRACE score or the MINAP-derived GRACE score.

COPD, chronic obstructive pulmonary disease; GRACE, Global Registry of Acute Coronary Events; MINAP, Myocardial Ischaemia National Audit Project.

**Figure 2:**
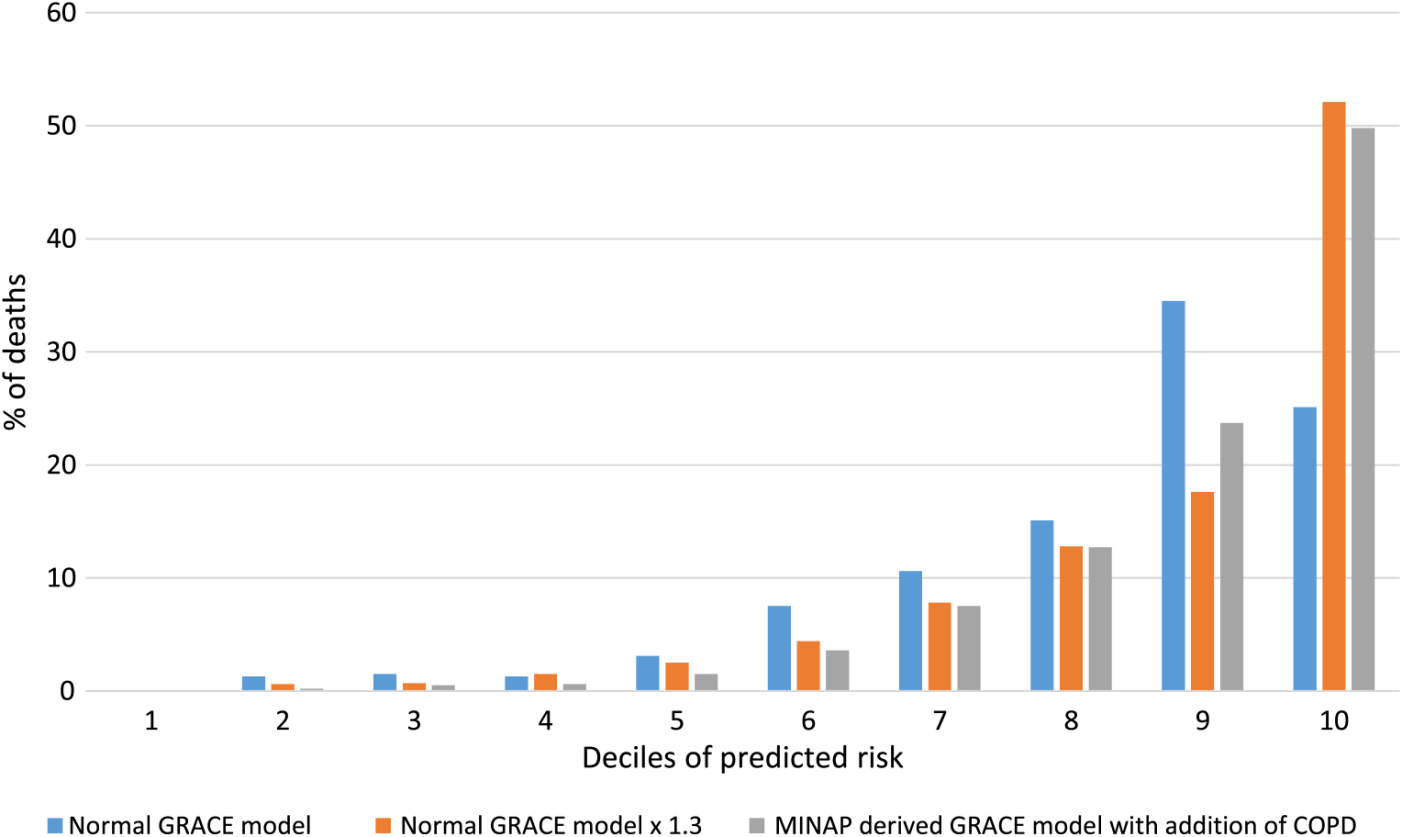
Proportion of deaths occurring in patients with COPD in each decile of predicted risk for the normal GRACE model, the GRACE model multiplied by 1.3 for patients with COPD, and the MINAP-derived model including COPD. COPD, chronic obstructive pulmonary disease; GRACE, Global Registry of Acute Coronary Events; MINAP, Myocardial Ischaemia National Audit Project.

The proportions of all deaths in patients with COPD in deciles of predicted risk for the normal GRACE model, the GRACE model multiplied by 1.3 and the MINAP-derived model including COPD are displayed in figure 2. The plot shows a steeper increase in the proportion of deaths in each decile for the GRACE model multiplied by 1.3, and the MINAP-derived model including COPD compared with the normal GRACE model, indicating better stratification for these two modifications. Observed mortality within GRACE score predicted risk groups for the normal GRACE model and for the modifications for patients with COPD and without COPD is presented in table 4.

**Table 4:** Observed mortality at 6 months for patients with COPD and non-COPD patients stratified by different versions of the GRACE score predicted risk of death

Normal GRACE score		
GRACE score predicted risk level	Observed mortality—non-COPD (%)	Observed mortality—COPD (%)
Low (<3%)	1.0	1.9
Med (3%–6%)	3.1	5.8
High (>6%)	18.4	23.3
**Normal GRACE score×1.3 for patients with COPD**		
**GRACE score predicted risk level**	**Observed mortality—non-COPD (%)**	**Observed mortality—COPD (%)**
Low (<3%)	1.0	1.3
Med (3%–6%)	3.1	3.8
High (>6%)	18.4	21.4
**MINAP-derived GRACE score**		
**GRACE score predicted risk level**	**Non-observed mortality—non-COPD (%)**	**Observed mortality—COPD (%)**
Low (<3%)	1.1	1.1
Med (3%–6%)	3.4	6.0
High (>6%)	20.6	25.2
**MINAP-derived GRACE score and COPD**		
**GRACE score predicted risk level**	**Observed mortality—non-COPD (%)**	**Observed mortality—COPD (%)**
Low (<3%)	1.1	1.4
Med (3%–6%)	3.7	4
High (>6%)	21.0	23.3

COPD, chronic obstructive pulmonary disease; GRACE, Global Registry of Acute Coronary Events; MINAP, Myocardial Ischaemia National Audit Project.

The findings for reclassification of risk levels after different model modifications are displayed in table 5. Compared with the normal GRACE score model, when patients with COPD were stratified into risk groups based on the multiplying the GRACE score predicted risk of death by 1.3, 33.9% of those classified as low risk (<3%) were reclassified as moderate risk (3%–6%), and 64.3% of those who were classified as moderate risk were reclassified as high risk (>6%). When stratified by type of ACS, the results were similar to the main analysis, with the exception of change in risk group after an STEMI in the MINAP-derived model including COPD (see online supplementary tables S2–S4). The NRI for adding COPD to the GRACE score model was 0.133 (p<0.001) indicating an improvement in classification of subjects when COPD is added to the model.

**Table 5:** Changes in level of risk for patients with COPD after modifications

	Multiplying risk by 1.3		
GRACE score predicted risk of death	Low risk (<3%)	Moderate risk (3%–6%)	High risk (≥6%)
Low risk (<3%)	4107 (66.1%)	2108 (33.9%)	0
Moderate risk (3%–6%)	0	2000 (35.7%)	3609 (64.3%)
High risk (>6%)	0	0	20 799 (100.0%)
	**Adding COPD into MINAP-derived GRACE model**		
**GRACE score predicted risk of death**	**Low risk (<3%)**	**Moderate risk (3%–6%)**	**High risk (≥6%)**
Low risk (<3%)	4635 (71.5%)	1582 (25.5%)	184 (3.0%)
Moderate risk (3%–6%)	681 (12.2%)	2792 (50.0%)	2117 (37.9%)
High risk (>6%)	15 (0.1%)	994 (4.8%)	19 527 (95.1%)

COPD, chronic obstructive pulmonary disease; GRACE, Global Registry of Acute Coronary Events; MINAP, Myocardial Ischaemia National Audit Project.

The findings from the multiple imputation analysis were similar to those from the main analysis, and are presented in the online supplementary table S5.

## Discussion

We found that GRACE scores for predicting risk of death at 6 months after ACS do not perform as well for people with COPD compared with those who do not have COPD. On average, patients with COPD had a 30% higher risk of death than non-COPD patients with the same GRACE score. To improve GRACE scores for patients with COPD, one option would be to respecify the GRACE model including COPD as a risk factor. Alternatively, multiplying GRACE score predicted risk of death by 1.3 for patients with COPD provides a very close approximation.

We found that, conditional on GRACE score predicted risk of death, patients with COPD had a higher risk of death than non-COPD patients, indicating that these scores underestimate the risks of death in those with COPD. One might argue that this might be true for any comorbidity; however, when we also estimated the relative risk of death comparing patients with diabetes with those without diabetes adjusted for GRACE score predicted risk of death, although we found an increased risk, this was much lower than for COPD. Although the relative risk of death for COPD might seem modest, this may have a large impact on patient treatment. Indeed, our results suggest that a large portion of patients with COPD would have been reclassified upwards in terms of level of risk if either of our suggested modifications (multiplying the risk for patients with COPD by 1.3 and adding COPD to the model) to the GRACE score had been used. We found that GRACE score predicted risk was closer to observed risk in those with COPD. The explanation for this is likely to be that for COPD and non-COPD patients with the same predicted risk of death, patients with COPD have always been at higher risk and observed mortality for all patients has fallen since GRACE scores were created such that they now by chance align well for those with COPD, rather than the GRACE score performing better estimated risk of death for those with COPD. This is consistent with our findings when we tabulated predicted and observed risk stratified by admission year. Although the GRACE score is the most accurate and widely used score for predicting risk of death after admission for ACS, others are in use. Clinicians should be aware that scores which use similar parameters are likely to underestimate risk of death for patients with COPD to a similar degree.

Our findings are an important contribution to discussion around the risk-treatment paradox. The paradox is that although people who are at highest risk of death after ACS are most likely to benefit from early aggressive therapy, they are the least likely to receive it.^19^ This may go some way in explaining why patients with COPD receive less in-hospital treatment after MI, such as in-hospital angiography after non-STEMI. Using risk scores and recommendations based on these to guide treatment decisions is one way to resolve this paradox. However, these risk scores must be able to predict risk of death well, they must be able to do this around levels of risk important for decision making, and they must do this for those at high risk of death.

A strength of our study is that it is large and representative of the national population, including all hospital admissions for ACS in England and Wales. A well as our complete case analysis, we also explored reasons for missing data and conducted a multiple imputation analysis. This further analysis did not change our conclusions. We calculated the proportion of patients with COPD who would have changed risk category as a result of the increase in predicted risk of death. This allowed us to demonstrate that although the relative risk of death adjusting for GRACE score predicted risk of death may seem modest, at the critical region of 0%–6% predicted risk of death, this could have resulted in a change in management for a substantial proportion of patients with COPD. One limitation of our study was that we used the National Institute for Health and Care Excellence-amended mini-GRACE score^17^ rather than the model including the Killip class. We used prescription of diuretics in hospital as a surrogate for acute heart failure. However, it is highly unlikely that the differences between patients with COPD and without COPD could be explained by this. In addition, recent work^17^ has shown that this GRACE score is a very good approximation to the full GRACE score, and the amended mini-GRACE score is being used in practice as it is now available on the GRACE 2.0 calculator.

There are several possible reasons why GRACE score predicted risk of death is not as accurate for patients with COPD. Our previous work showed that the relative risk of death after MI for patients with COPD is greater after non-STEMIs than STEMIs,^14^ and non-STEMIs will be scored lower than STEMIs, all other things being equal. In addition, the effect of COPD on risk of death after MI was greater for younger patients with COPD, and younger people will be scored lower on average. In the development of the GRACE score, although several clinical characteristics, including diabetes, hypertension and hyperlipidaemia were tested for inclusion as risk factors, COPD was not.^20^ Although some of the increased risk of death may be due to differences in treatment, others have concluded that the GRACE score maintains its predictive ability even in groups with different treatment.^21^ In addition, among a wide range of in-hospital treatments tested, none entered the GRACE score model as predictors of death.^20^ Previous work has investigated the performance of the GRACE score in other high risk groups such as people with diabetes and with chronic renal failure.^22^ However, this work only assessed the C-statistics in these groups, and did not involve assessing the GRACE score in those with COPD. Our findings have important clinical implications for the care of patients with COPD after admission to hospital for ACS. Multiplying the GRACE score predicted risk of death by 1.3 for patients with COPD would mean that 34% of people with COPD would move from being classified as low risk to moderate risk (<3% to 3%–6%). These changes have important implications as recommendations for treatment after non-STEMI and unstable angina are based on classification as moderate or high predicted risk of death. This is particularly relevant as it is known that patients with COPD are more likely to present with a non-STEMI than non-COPD patients and that the effect of COPD on risk of death after MI is highest in non-STEMIs, and after adjusting for patient characteristics, they are less likely to receive early invasive treatment after a non-STEMI compared with non-COPD patients.^14^ ^23^

## Conclusions

The GRACE score predicted risk of death after ACS does not predict risk of death for people with COPD as well as they do for those who do not have COPD, and underestimates risk of death for this group. When future versions of the GRACE score model are created, those developing the scores may want to include COPD as a risk factor for death. Clinicians should multiply GRACE score predicted risk of death by 1.3 to obtain a more accurate prediction. Using this rule would mean that one third of patients with COPD previously considered to be low risk should be considered moderate risk and would be considered for more aggressive early treatment under current guidelines for non-STEMI and unstable angina.

**Key messages**

**What is already known on this subject?**

- Despite being at higher risk of death following admission for acute coronary syndromes, patients with chronic obstructive pulmonary disease (COPD) are less likely to receive investigation and treatment than those without COPD and this difference may explain some of the difference in mortality. It is recommended that those at moderate (3%–6%) or high (>6%) Global Registry of Acute Coronary Events (GRACE) score predicted risk of death at 6 months after admission to hospital for non-ST-segment elevation myocardial infarction or unstable angina receive earlier aggressive investigation and treatment.

**What might this study add?**

- This nationwide multicentre study involving 481 849 hospital admissions demonstrates that GRACE scores underestimate risk of death after acute coronary syndromes for those with COPD. This study also found that multiplying the predicted risk of death for those with COPD by 1.3 provides a better approximation for their risk of death.

**How might this impact on clinical practice?**

- Using a more accurate estimate of risk of death for those with COPD after admission for acute coronary syndromes one third of patients with COPD previously categorised as low risk would be reclassified as moderate risk, and therefore would be eligible for earlier, more aggressive investigation and treatment.
