# Supplementary material for: Predicting mortality after acute coronary syndromes in people with chronic obstructive pulmonary disease
Source: Heart. 2016 May 13;102(18):1442–8. doi: 10.1136/heartjnl-2016-309359 (PMC5013109; doi:10.1136/heartjnl-2016-309359)
Supplement: Supplementary data [file heartjnl-2016-309359supp.pdf]

Supplementary material

Table S1 Predicted and observed mortality using normal GRACE model stratified by year of admission.

	**2006-2008**			**2009-2010**			**2012-2013**		
**GRACE predicted risk decile**	**Average predicted mortality (%)**	**Observed mortality - non-COPD (%)**	**Observed mortality – COPD (%)**	**Average predicted mortality (%)**	**Observed mortality - non-COPD (%)**	**Observed mortality – COPD (%)**	**Average predicted mortality (%)**	**Observed mortality - non-COPD (%)**	**Observed mortality – COPD (%)**
1	1.3	0.8	0.8	1.3	0.5	0.7	1.3	0.6	1.1
2	2.5	1.6	3.2	2.5	1.3	2.4	2.5	1.0	1.7
3	4.0	3.0	5.9	4.0	2.5	3.8	4.0	1.7	4.5
4	5.0	3.9	7.6	5.0	3.2	6.0	5.0	2.5	6.0
5	6.5	5.5	9.2	6.5	4.6	7.3	6.4	3.3	6.2
6	8.9	8.6	13.3	8.9	7.2	12.6	8.9	5.4	10.4
7	12.4	12.4	18.7	12.4	11.1	17.9	12.4	8.4	13.7
8	17.2	18.8	24.8	17.2	16.8	22.0	17.2	14.4	18.4
9	26.6	30.3	35.3	26.6	27.5	32.5	26.6	23.0	27.8
10	48.5	46.6	50.8	48.3	44.5	48.0	48.7	39.8	43.6

Table S2 Changes in level of risk for COPD patients after modifications after a STEMI

	**Multiplying risk by 1.3**		
**GRACE score predicted risk of death**	**Low risk (<3%)**	**Moderate risk (3-6%)**	**High risk (≥6%)**
**Low risk (<3%)**	791 (60.4%)	519 (39.6%)	0 (0.0%)
**Moderate risk (3-6%)**	0 (0.0%)	499 (32.8%)	1,022 (67.2%)
**High risk (≥6%)**	0 (0.0%)	0 (0.0%)	5,564 (100.0%)
	**Adding COPD into MINAP derived GRACE model**		
**GRACE score predicted risk of death**	**Low risk (<3%)**	**Moderate risk (3-6%)**	**High risk (≥6%)**
**Low risk (<3%)**	1,171 (89.5%)	138 (10.5%)	7 (0.5%)
**Moderate risk (3-6%)**	423 (27.8%)	909 (59.8%)	188 (12.4%)
**High risk (≥6%)**	10 (0.2%)	587 (10.7%)	4,902 (89.1%)

Table S3 Changes in level of risk for COPD patients after modifications after a non-STEMI

	**Multiplying risk by 1.3**		
**GRACE score predicted risk of death**	**Low risk (<3%)**	**Moderate risk (3-6%)**	**High risk (≥6%)**
**Low risk (<3%)**	1,742 (65.3%)	924 (34.7%)	0 (0.0%)
**Moderate risk (3-6%)**	0 (0.0%)	912 (36.2%)	1,611 (63.9%)
**High risk (≥6%)**	0 (0.0%)	0 (0.0%)	10,603 (100.0%)
	**Adding COPD into MINAP derived GRACE model**		
**GRACE score predicted risk of death**	**Low risk (<3%)**	**Moderate risk (3-6%)**	**High risk (≥6%)**
**Low risk (<3%)**	1,909 (71.7%)	698 (26.2%)	55 (2.1%)
**Moderate risk (3-6%)**	184 (7.3%)	1,227 (48.8%)	1,105 (43.9%)
**High risk (≥6%)**	4 (0.0%)	289 (2.8%)	10,176 (97.2%)

Table S4 Changes in level of risk for COPD patients after modifications after unstable angina

	Multiplying risk by 1.3		
**GRACE score predicted risk of death**	**Low risk (<3%)**	**Moderate risk (3-6%)**	**High risk (≥6%)**
**Low risk (<3%)**	1,569 (70.3%)	664 (29.7%)	0 (0.0%)
**Moderate risk (3-6%)**	0 (0.0%)	589 (37.7%)	972 (62.3%)
**High risk (≥6%)**	0 (0.0%)	0 (0.0%)	4, 623 (100.0%)
	**Adding COPD into MINAP derived GRACE model**		
**GRACE score predicted risk of death**	**Low risk (<3%)**	**Moderate risk (3-6%)**	**High risk (≥6%)**
**Low risk (<3%)**	1,362 (61.1%)	746 (33.5%)	122 (5.5%)
**Moderate risk (3-6%)**	74 (4.8%)	656 (42.2%)	824 (53.0%)
**High risk (≥6%)**	1 (0.0%)	118 (2.6%)	4,449 (97.4%)

Multiple imputation and missing data

There were significant levels of missing data for creatinine (40%), systolic blood pressure (11%), and heart rate (11%). Missingness was associated with year of event, and was greatly reduced in events after 2008 (<10% missingness for all three variables).

As an additional analysis, we multiply imputed [1] values for serum creatinine, heart rate, and systolic blood pressure where these were missing. Predictor variables were all other GRACE score variables, COPD status and death at 6 months. As the missing variables were all continuous, we performed multiple imputation using multivariate normal regression using the "mi impute mvn" command in Stata 14.1 MP. We imputed 30 additional datasets and used these to test our modifications to the GRACE score estimated probability of death. We did this using logistic regression to compare mortality at 6 months after admission

The findings from the multiple imputation analysis indicated that GRACE scores underestimate the risk of death for people with COPD, that adding COPD to the GRACE score model would fix this problem, and that multiplying GRACE score predicted probability of death by 1.3 was a good approximation to adding COPD to the model.

Table S5 Results of multiple imputation analysis

**GRACE model or modification**	**OR (95% CI)**
Normal GRACE model	1.39 (1.36-1.43)
Normal GRACE model x 1.3 for COPD patients	0.95 (0.92-0.98)
MINAP derived model	1.34 (1.30-1.39)
MINAP derived model with smoking	1.42 (1.36-1.47)
MINAP derived model with COPD	1.02 (0.99-1.06)

References

1. Carpenter JR, Kenward MG. Multiple imputation and its application. New York: Wiley, 2013.
